# Novel HMO-Glasses with Sb_2_O_3_ and TeO_2_ for Nuclear Radiation Shielding Purposes: A Comparative Analysis with Traditional and Novel Shields

**DOI:** 10.3390/ma14154330

**Published:** 2021-08-03

**Authors:** Ghada ALMisned, Huseyin Ozan Tekin, Shams A. M. Issa, Miray Çelikbilek Ersundu, Ali Erçin Ersundu, Gokhan Kilic, Hesham M. H. Zakaly, Antoaneta Ene

**Affiliations:** 1 Department of Physics, College of Science, Princess Nourah Bint Abdulrahman University, Riyadh 11671, Saudi Arabia; gaalmisned@pnu.edu.sa; 2 Department of Medical Diagnostic Imaging, College of Health Sciences, University of Sharjah, Sharjah 27272, United Arab Emirates; tekin765@gmail.com; 3 Medical Radiation Research Center (USMERA), Uskudar University, Istanbul 34672, Turkey; 4 Physics Department, Faculty of Science, University of Tabuk, Tabuk 71451, Saudi Arabia; shams_issa@yahoo.com; 5 Physics Department, Faculty of Science, Al-Azhar University, Assiut 71524, Egypt; 6 Glass Research and Development Laboratory, Department of Metallurgical and Materials Engineering, Faculty of Chemical and Metallurgical Engineering, Yildiz Technical University, Istanbul 34220, Turkey; miraycelikbilek@gmail.com (M.Ç.E.); ersundu@gmail.com (A.E.E.); 7 Department of Physics, Faculty of Science and Letters, Eskisehir Osmangazi University, Eskisehir 26040, Turkey; gkilic@ogu.edu.tr; 8 Institute of Physics and Technology, Ural Federal University, 620000 Ekaterinburg, Russia; 9 INPOLDE Research Center, Department of Chemistry, Faculty of Sciences and Environment, Physics and Environment, Dunarea de Jos University of Galati, 47 Domneasca Street, 800008 Galati, Romania

**Keywords:** HMO glasses, radiation shielding, TeO_2_, Sb_2_O_3_

## Abstract

The radiation shielding characteristics of samples from two TeO_2_ and Sb_2_O_3_-based basic glass groups were investigated in this research. TeO_2_ and Sb_2_O_3_-based glasses were determined in the research as six samples with a composition of 10WO_3_-(x)MoO_3_-(90 − x)(TeO_2_/Sb_2_O_3_) (x = 10, 20, 30). A general purpose MCNPX Monte Carlo code and Phy-X/PSD platform were used to estimate the radiation shielding characteristics. Accordingly, the linear and mass attenuation coefficients, half value layer, mean free path, variation of the effective atomic number with photon energy, exposure and built-up energy factors, and effective removal cross-section values were determined. It was determined that the results that were produced using the two different techniques were consistent. Based on the collected data, the most remarkable findings were found to be associated with the sample classified as T80 (10WO_3_ + 10MoO_3_ + 80TeO_2_). The current study showed that material density was as equally important as composition in modifying radiation shielding characteristics. With the T80 sample with the greatest density (5.61 g/cm^3^) achieving the best results. Additionally, the acquired findings were compared to the radiation shielding characteristics of various glass and concrete materials. Increasing the quantity of MoO_3_ additive, a known heavy metal oxide, in these TeO_2_ and Sb_2_O_3_-based glasses may have a detrimental impact on the change in radiation shielding characteristics.

## 1. Introduction

Current findings have conclusively demonstrated that glass-based materials have a wide range of applications in various technology and industry sectors. In addition to this, working with glasses is quite flexible, both in terms of development and structural flexibility. This enables researchers to identify the most appropriate structure for their intended function by altering a range of different glass designs, which they can then test. However, each glass manufacturing process necessitates developing a unique set of characterization methods, based on experimental and modeling approaches, to ensure that the results are understandable and acceptable for the purposes for which they are designed. Among the different types of glasses, heavy metal oxide glasses (HMO) have received a lot of interest lately because of their low phonon characteristics [1,2]. Glasses with more than 50% mol percent of a heavy metal cation are heavy metal oxide glasses. The glasses TeO_2_, Sb_2_O_3_, Bi_2_O_3_, and PbO are repetitive members of the HMO glass family. These glasses are excellent photonic matrices, due to their larger transparency interval that covers the visible to mid-infrared range, better non-linear optical characteristics, greater solubility of rare-earth ions, and lower phonon energies than conventional silicate, borate, and phosphate glasses. Apart from their excellent thermal, mechanical, and chemical durability, heavy metal oxide glasses have outstanding optical and electrical characteristics, including a high refractive index and dielectric constant. Due to their outstanding properties, heavy metal oxide glasses are excellent candidates for many optoelectronic applications, including fiber optics, lasers, and sensors. However, material density (g/cm^3^) is a significant feature of candidate materials for gamma radiation shielding applications. The literature reviewed showed that HMO glasses had been evaluated in terms of their gamma-ray attenuation properties, thanks to their high material density. In this regard, Celikbilek Ersundu et al. have performed different types of studies on different types of fabricated HMO glasses with nominal compositions of K_2_O-WO_3_-TeO_2_ and ZnO-MoO_3_-TeO_2_ [3,4]. According to their findings, K30W60T10 and Z10M10T80 glasses were reported as the most effective shielding glasses, owing to their higher performance against an ionizing gamma-ray. Our review showed that the similarity between these two glass samples is that they have the maximum TeO_2_ ratio in their structure. Additionally, a study of the literature revealed that many studies examined HMO reinforced glasses. For instance, Al-Hadeethi and Sayyed have analysed some HMO doped borosilicate glasses by using Geant4 simulation code [5]. Their findings showed that the inclusion of the three dopants, such as Bi_2_O_3_, BaO, and TiO_2_ resulted in a drop in the HVL (the thickness of the material at which the intensity of radiation entering it is reduced by one half), which resulted in an improvement in the attenuation performance of the studied HMO glasses. In another study, D’Souza et al. investigated the effect of Bi_2_O_3_ on the structural, optical, mechanical, radiation shielding, and luminescence characteristics of borosilicate glasses containing HMO [6]. Their results indicated that with repeated additions of Bi_2_O_3_, the gamma-ray shielding capacity rose, but the neutron attenuation capacity dropped. The results of our earlier investigations have prompted us to do further research on HMO glasses that incorporate more comprehensive ideas. Following a successful search of the literature, six different HMO glasses with various substitutions were identified and successfully tested, to better understand the potential effects of substituted heavy metal oxides, such as TeO_2_ and Sb_2_O_3_ [7]. The current investigation aims to evaluate the direct contributions of TeO_2_ and Sb_2_O_3_ on HMO glasses, in terms of gamma-ray attenuation properties. In addition to nuclear radiation (gamma and neutron) shielding characteristics, the synergistic effects of the substitutions on nuclear radiation shielding behaviors will be discussed. Additionally, the data will be compared to certain existing shielding materials and shielding glasses to determine whether the investigated glasses are potentially superior to traditional and disadvantageous shields.

## 2. Materials and Methods

Six distinct HMO glasses with a range of substitutions were found and successfully tested using MCNPX Monte Carlo code [8] and Phy-X/PSD [9], in order to obtain a better knowledge of the possible impacts of substituted heavy metal oxides, such as TeO_2_ and Sb_2_O_3_. As a result, we sought to study several forms of HMO glasses, based on various distinct principles [7], as follows.

- 10WO_3_ + 10MoO_3_ + 80TeO_2_;
- 10WO_3_ + 20MoO_3_ + 70TeO_2_;
- 10WO_3_ + 30MoO_3_ + 60TeO_2_;
- 10WO_3_ + 10MoO_3_ + 80Sb_2_O_3_;
- 10WO_3_ + 20MoO_3_ + 70Sb_2_O_3_;
- 10WO_3_ + 30MoO_3_ + 60Sb_2_O_3_.

**Figure 1:**
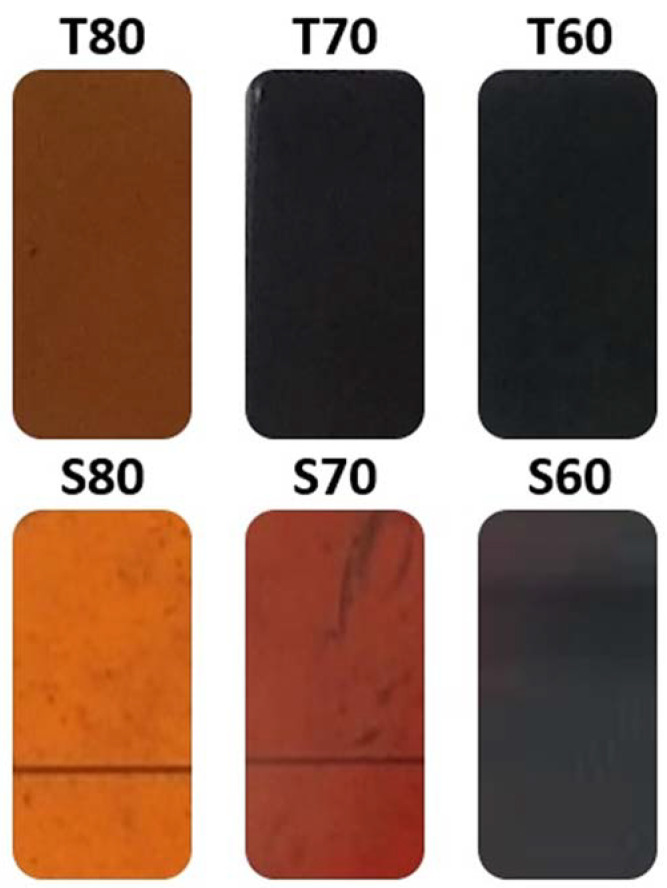
Physical appearances of T and S glasses.

As shown in Table 1, six different HMO glasses were characterized using different in silico methods, considering their elemental properties and densities. Figure 1 shows the physical appearances of the T80, T70, T60, S80, S70, and S60 HMO glasses.

### 2.1. Simulation

**Figure 2:**
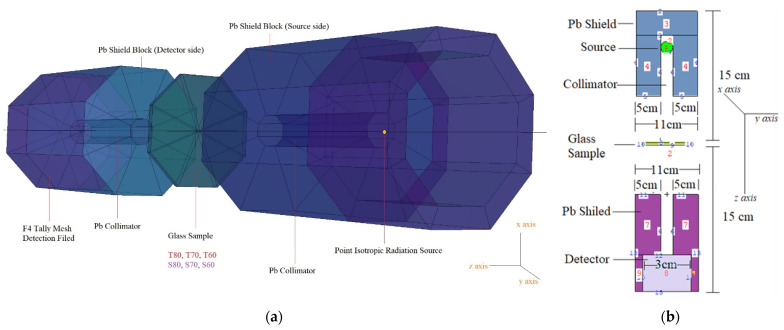
MCNPX simulation setup used for gamma-ray transmission simulations. (**a**) A direct screenshot from the MCNPX Visual Editor VE X_22S. (**b**) The suggested MCNPX simulation setup for evaluating the gamma-ray transmission capacities of glasses.

Figure 2a shows the utilized MCNPX design as a direct screenshot from the MCNPX Visual Editor. To calculate the linear attenuation coefficients (*µ*) to be obtained as a result of gamma transmission, a point isotropic radiation source, HMO glass, and a detection field were defined. MCNPX is a general purpose Monte Carlo technique that has been utilized for the determination of mass attenuation coefficients. First, the INPUT file to be used for the MCNPX (version 2.7.0) [8] code was created by defining the materials and the overall gamma transmission setup. For this aim, we have defined the input data for MCNPX using the following fundamental components.

- Cell-Card;
- Surface-Card;
- Information about the source.

The glass specimens’ elemental mass fractions as well as densities (g/cm^3^) were used to create models (in grams per cubic centimeter). The glass specimen (T or S) is cylindrical in shape with a radius of 5 cm, and is composed of transparent glass. As a result of this development, the boundaries of cell cards were saturated with the material properties necessary for their production (i.e., elemental mass fraction and material density). In Figure 2b, the suggested MCNPX simulation setup for evaluating the gamma-ray transmission capacities of glasses, such as T and S, is shown in two dimensions. To record the quantity of attenuated (secondary) gamma-rays, T and S glasses were linked to the opposite side of the detector field (F4 Tally Mesh). The F4 is advantageous for determining the mean photon flux within a point or cell. Finally, it is worth mentioning that a total of 10^8^ particles with varying photon energies were administrated for each glass sample (i.e., between 0.015–15 MeV). When all simulations were conducted, the MCNPX output had a relative error rate of less than 1%.

### 2.2. Studied Properties

After determining the linear attenuation coefficients (*µ*) in the preceding section, many additional gamma radiation attenuation parameters were computed. First, we determined the mass attenuation coefficients (*µ_m_*) of the T and S glasses using Equation (1) [10].

(1) $$\mu_{m}=\frac{\mu }{\rho }$$

where *μ* is the linear attenuation coefficient, and *ρ* is the density. Next, half value layer (T_1/2_) values of T and S glasses were also determined. The term T_1/2_ has a decisive role in implementing very critical radiation protection measures. This value offers critical information regarding the thicknesses at which a shielding material may effectively halve the intensity of a received gamma-ray. Further, other critical gamma-ray shielding properties, such as mean free path (λ), effective atomic numbers (Z_eff_) against gamma-ray attenuation, exposure, and energy absorption build-up factors (EBF and EABF) (defined as the photon build-up factor in which the quantity of interest is the exposure and the detector response function of the absorption in air, and absorbed or deposited energy in the medium considered) were determined in a gamma-ray energy range of 0.015–15 MeV. On the other hand, we intended to assess the attenuation performances of the T and S glasses against fast neutrons. Therefore, effective removal cross-section values of T60, T70, T80, S60, S70, and S80 glasses against fast neutrons (Σ_R_) were determined. Detailed information about the studied parameters can be found in THE literature elsewhere [11,12,13,14]. In addition to determining parameters, HVL values of the T80 sample were compared to those of many shielding glasses available in the literature, and various types of concrete. The details of the extended comparison are presented below.

- Group 1: TZNG-A [15], TZNG0.5 [16], Gd10 [17], Gd15 [18], PNCKM5 [19], C25 [20], SCNZ7 [21].
- Group 2: Ordinary Concrete (OC), HSC, ILC, BMC, IC, SSC [22].

## 3. Results and Discussion

**Figure 3:**
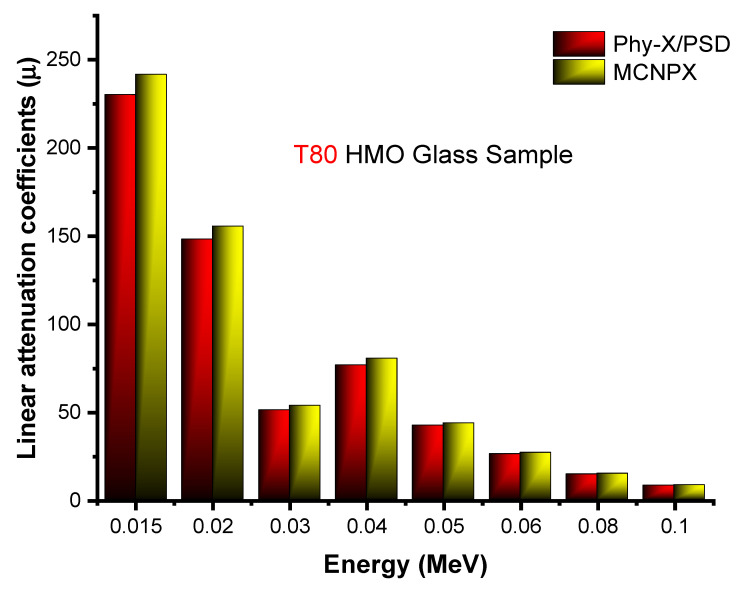
Comparison of linear attenuation coefficient values, obtained from MCNPX and Phy-X/PSD at a low gamma-ray energy region.

To identify the gamma-ray shielding properties of T60, T70, T80, S70, S80, and S90 glasses, some essential parameters have been determined. Meanwhile, we administered two effective tools, MCNPX and Phy-X/PSD, to determine the linear attenuation coefficients (*µ*) of all the glasses examined. Notably, the linear attenuation coefficients obtained using Phy-X/PSD and MCNPX (2.7.0) were very similar. Figure 3 compares the linear attenuation coefficients derived using the MCNPX Monte Carlo algorithm and Phy-X/PSD for the T80 glass sample. In general, our results indicated that relative differences ranged between 1.17 and 2.9 percent across all photon intensities. Overall, both outcomes were reported with similar linear attenuation coefficient values. However, small differences in specific energy fields have been identified. This is directly related to the nature of the tools used, since MCNPX is a Monte Carlo-based radiation transport method that requires user definition at various phases of the procedure, as outlined previously. In comparison, Phy-X/PSD is a web-based tool that only needs information on the material’s structure, density, and energy. Consequently, several other dissimilarities are almost certainly due to various factors, including the number of dispersed gamma-rays entering the detecting field, narrow beam shape, cross-section libraries, physics-lists used, hardware efficiency, and the CPU attributes of the computer systems used.

**Figure 4:**
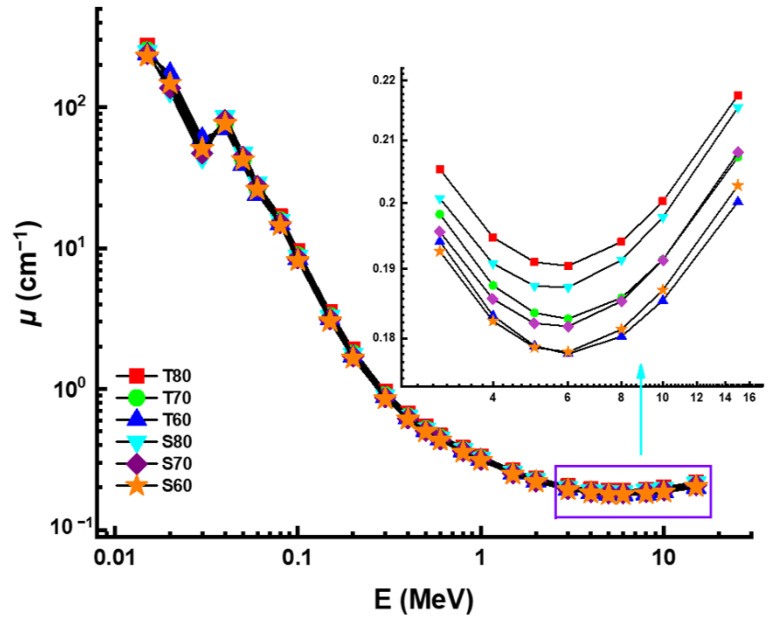
Variation of linear attenuation coefficient (*µ*) against photon energy for all glasses.

**Figure 5:**
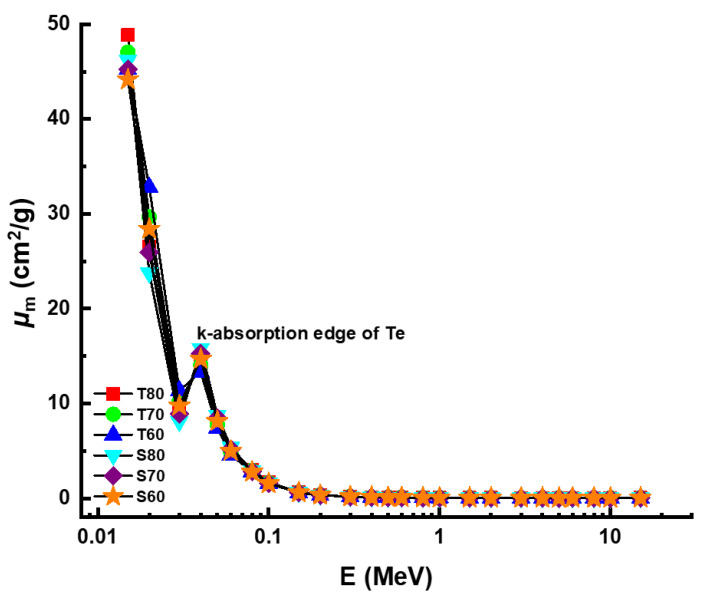
Variation of mass attenuation coefficient (*µ_m_*) against photon energy for all glasses.

Figure 4 demonstrates the variable trend of linear attenuation coefficient (*µ*) values, as a function of incident photon energy (MeV). As shown by the graph, linear attenuation coefficients dropped as energy increased. Nonetheless, a rapid decrease was used to achieve the low energy zone, dominated by photoelectric activity. Linear attenuation coefficients decreased in the mid-energy region, due to the primary interaction between incoming photons and attenuator glass samples, known as Compton scattering. However, our findings on *µ* values showed that the T80 sample with the maximum TeO_2_ reinforcement in the glass composition had the highest *µ* values at all administrated photon energies. For example, *µ* values were reported as 274.1192 cm^−1^, 254.6075 cm^−1^, 239.2530 cm^−1^, 250.7937 cm^−1^, 239.2946 cm^−1^ and 230.1126 cm^−1^ for T80, T70, T60, S80, S70, and S60 at 0.015 MeV, respectively. Initial results showed that the T80 sample also had the highest material density, which was reported as 5.61 g/cm^3^. The T80 sample’s superiority may explain this, not only in terms of density but also in terms of the elemental characteristics of TeO_2_ and Sb_2_O_3_. On the other hand, another density-independent gamma-ray attenuation quantity, known as mass attenuation coefficients (*µ_m_*), was also seen to follow a similar pattern. The resulting mass attenuation coefficients changed from 0.015 MeV to 15 MeV in response to the gamma-ray energy, as shown in Figure 5. 

**Figure 6:**
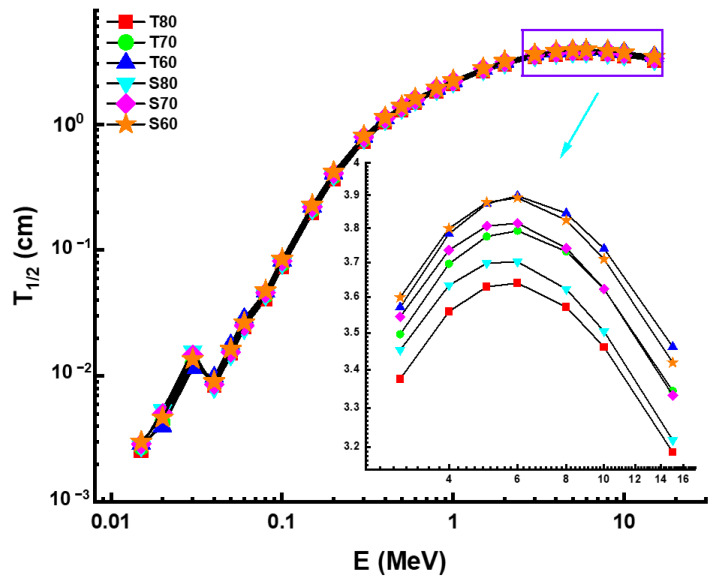
Variation of half value layer (T_1/2_) against photon energy for all glasses.

The dominance of the different interaction types were similarly determined for the same energy ranges as the previous findings. The T80 sample, in particular, showed the highest mass attenuation coefficients. According to this result, the chemical structure of the T80 sample, combined with the greatest concentration of TeO_2_ additive, is superior in the T and S glass family. When it comes to adopting very important radiation protection measures, the half value layer (T_1/2_) is essential [23]. Important information may be gained from this quantity, including the thicknesses at which a shielding material can effectively reduce the intensity of gamma-rays reflected off it by half. The variation of T_1/2_ as a function of increasing photon energy is demonstrated in Figure 6. 

**Figure 7:**
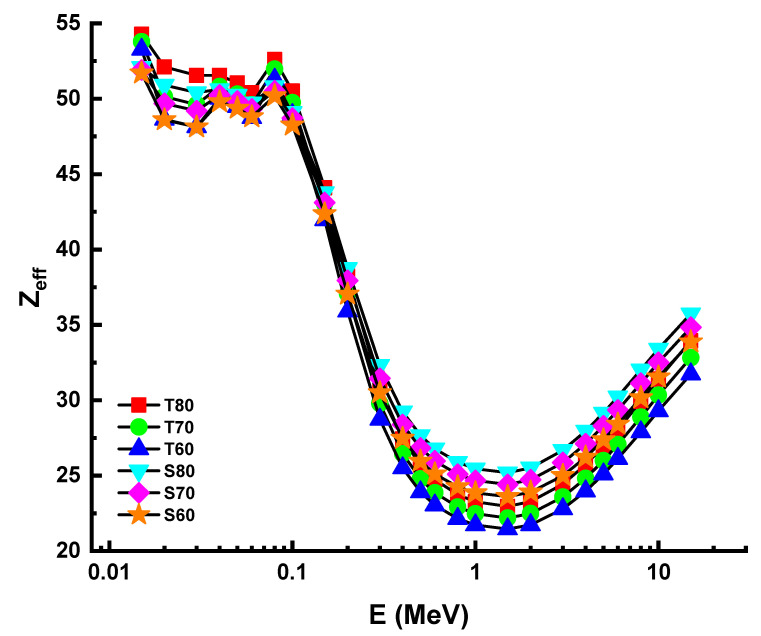
Variation of effective atomic number (Z_eff_) against photon energy for all glasses.

One may anticipate that the lowest T_1/2_ values for a material with the highest *µ* values among the shielding materials will be examined. This was confirmed for the HMO glasses examined, where the lowest T_1/2_ values were likewise recorded for the T80 sample. The change in the effective atomic number (Z_eff_) values as a function of photon energy is seen in Figure 7. In general, it is believed that elements having a more significant atomic number are more efficient at attenuating gamma-rays [24,25]. Although the T80 sample exhibited the highest Z_eff_ values at the energy levels examined, our data indicate that the difference between the two samples is not statistically significant. This may be due to the considerable variation in the molar (percent) concentrations of TeO_2_ and Sb_2_O_3_ substitutions.

**Figure 8:**
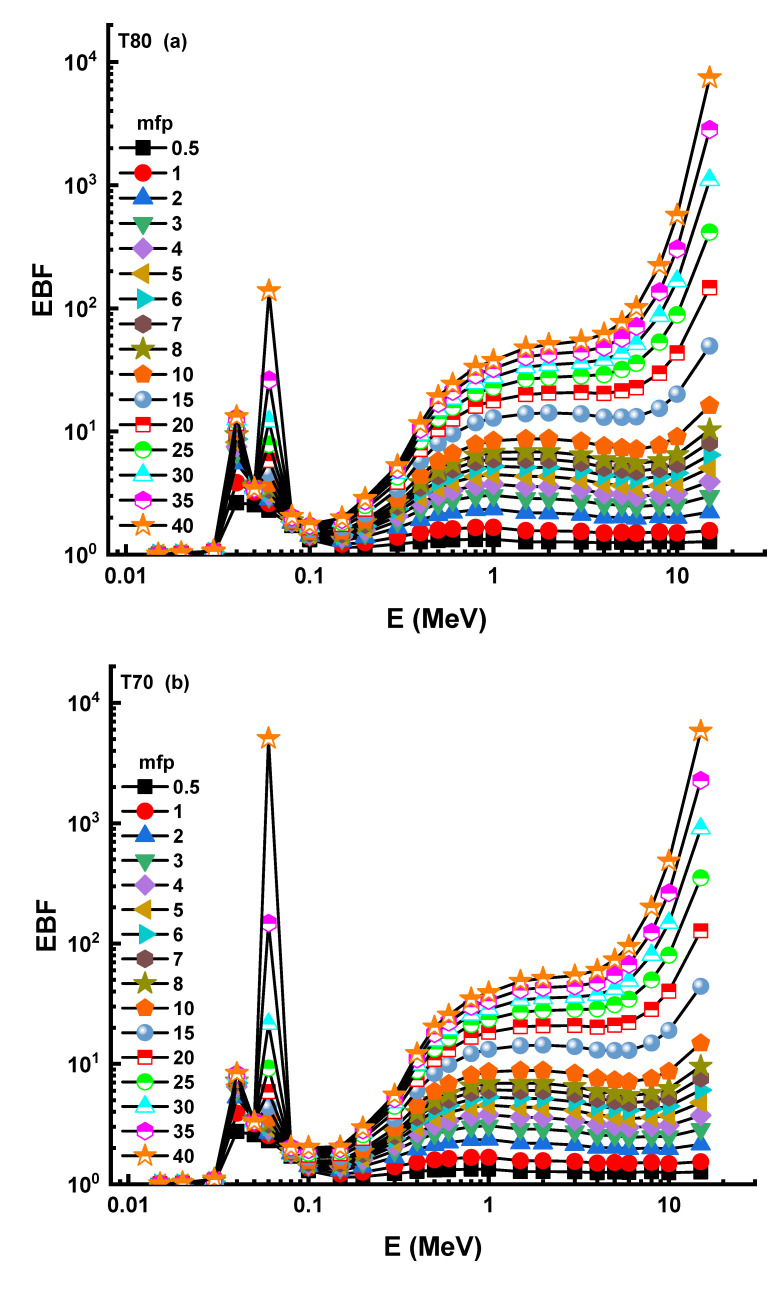

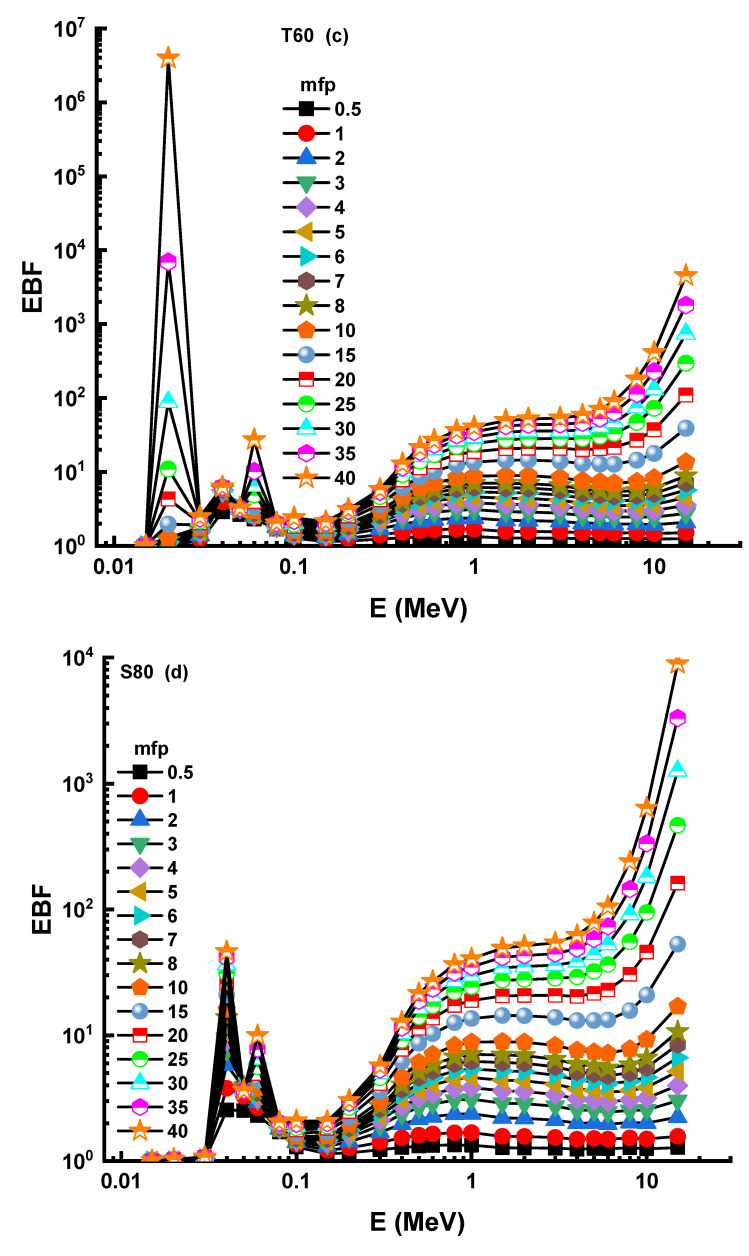

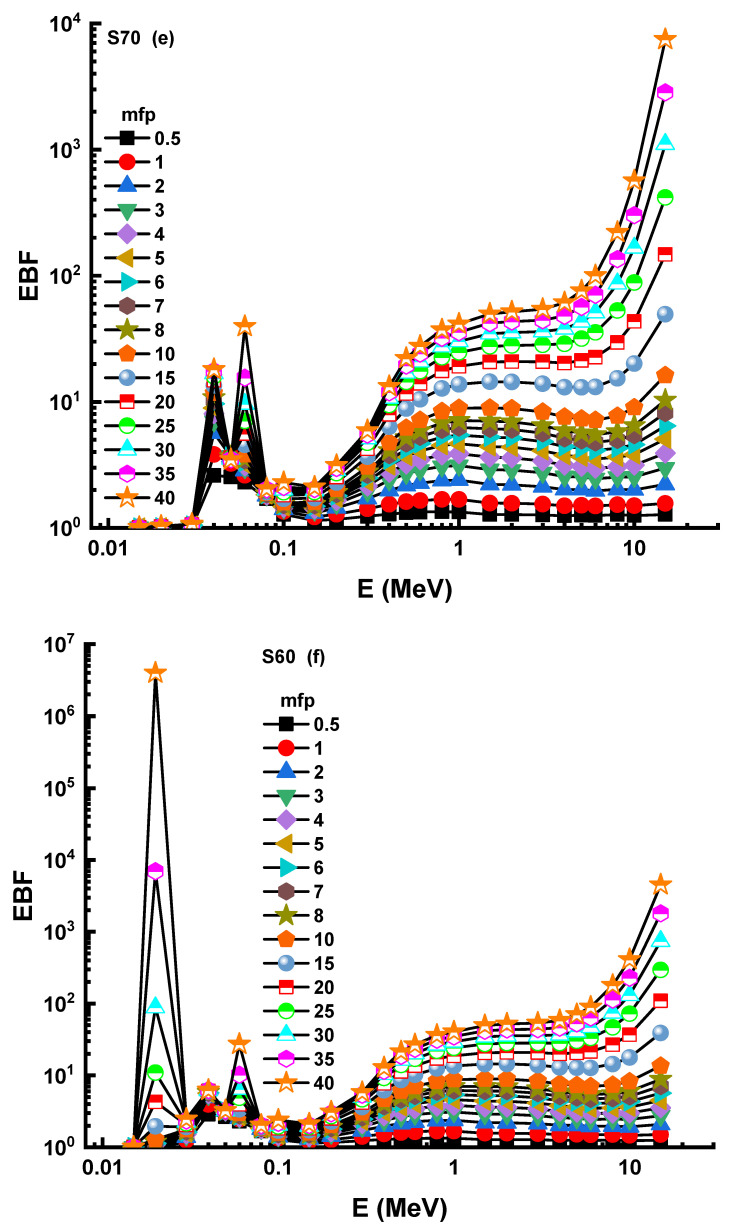
(**a**–**f**) Variation of exposure buildup factor (EBF) against photon energy for all glasses.

**Figure 9:**
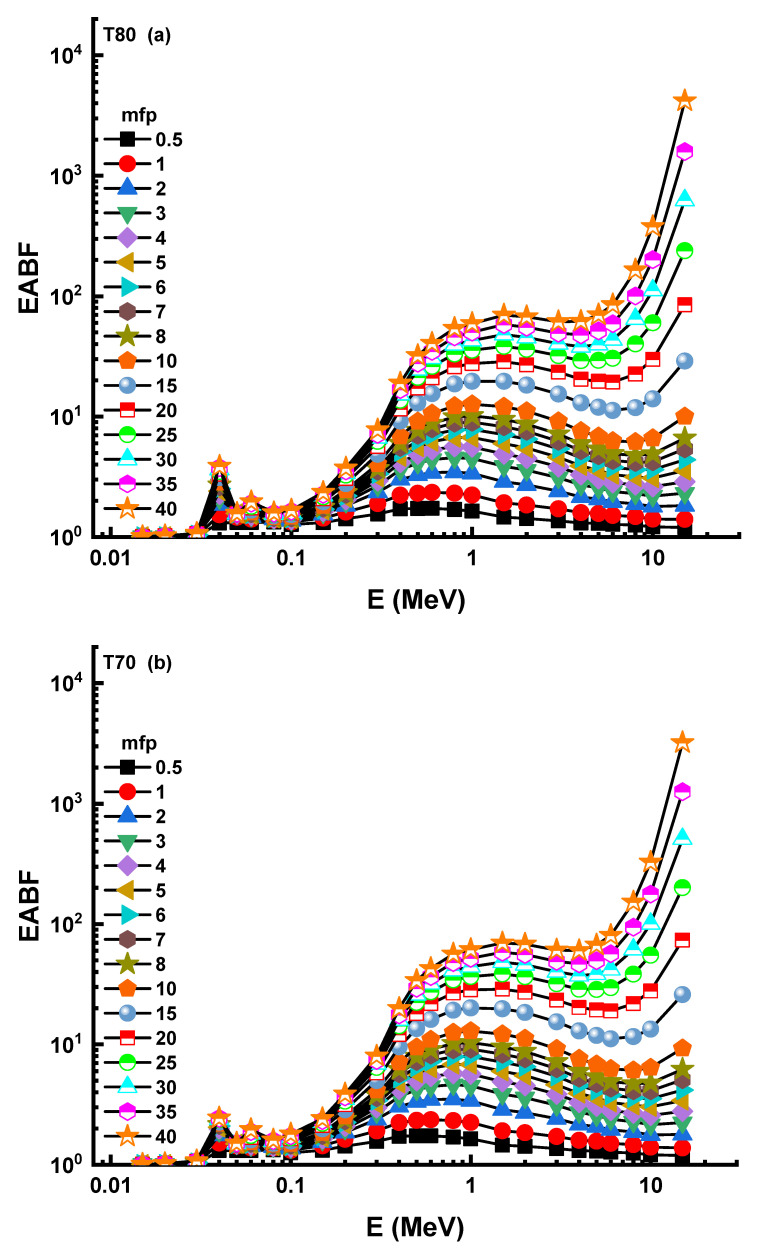

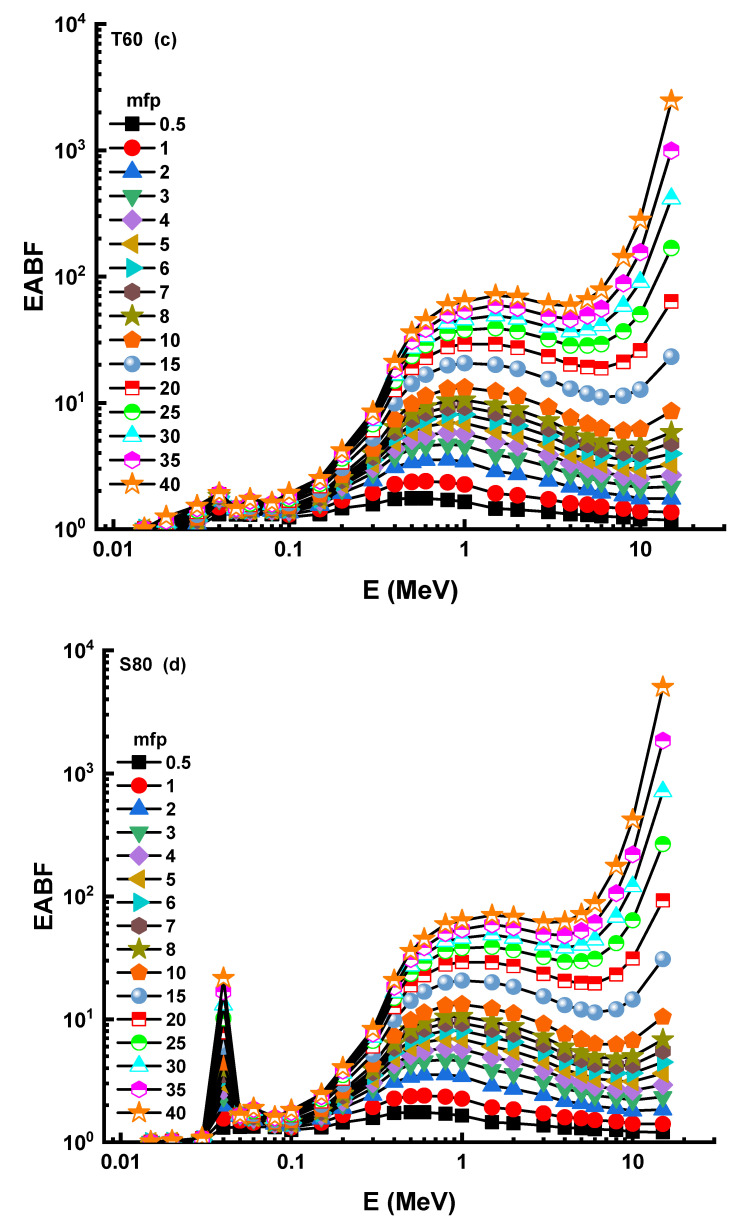

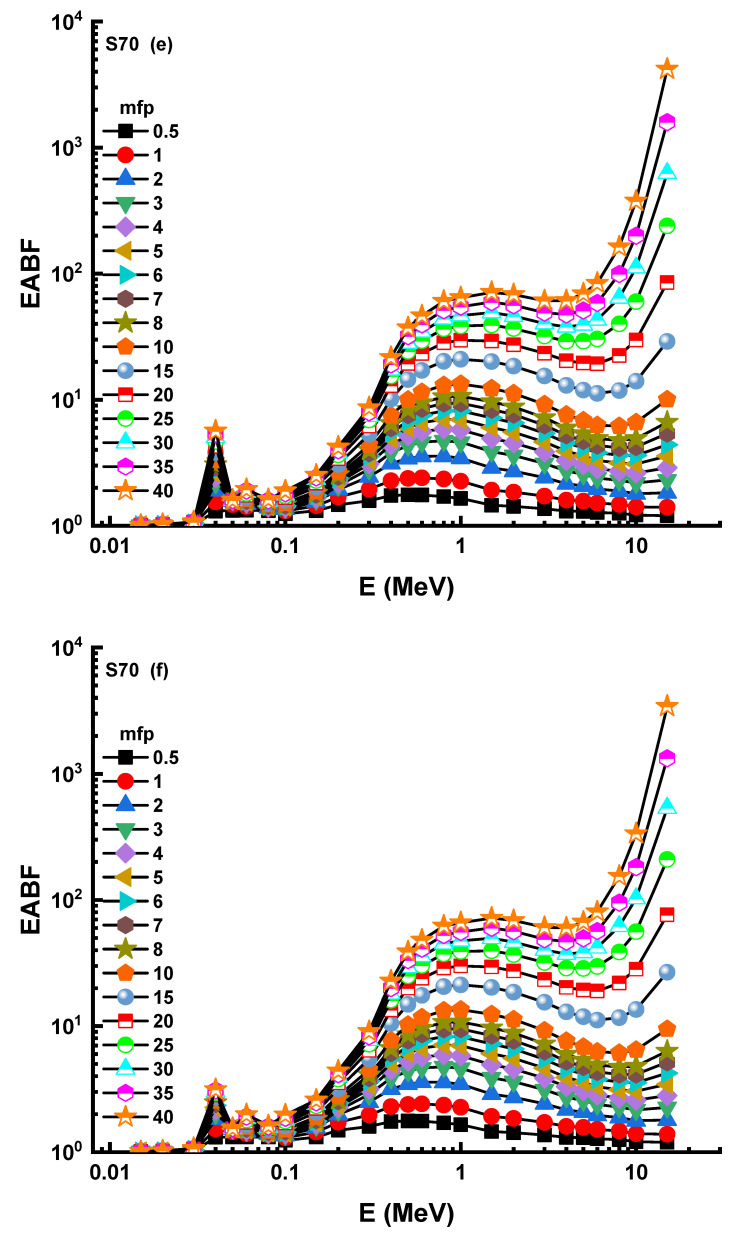
(**a**–**f**) Variation of energy absorption buildup factor (EABF) against photon energy for all glasses.

The nuclear photon build-up factor must be considered when assessing nucleonic data, such as radiation shielding and dosimetry. Colliding photons contribute to the goal in proportion to the rise in the build-up factor. The geometry progressive (G-P) technique (Supplementary Tables S1–S6) was used to calculate these variables in this research, yielding both the exposure build-up factor (EBF) and the energy absorption build-up factor (EABF). As a consequence, Figure 8 and Figure 9 show the variation of EBF and EABF with respect to the received photon energies for all T and S glass samples, with penetration depths from 0.5 to 40 mfp, respectively. When gamma-rays are absorbed, the majority of absorption occurs in the low (photoelectric dominant) and high energy bands, where relatively little particle accumulation occurs. 

**Figure 10:**
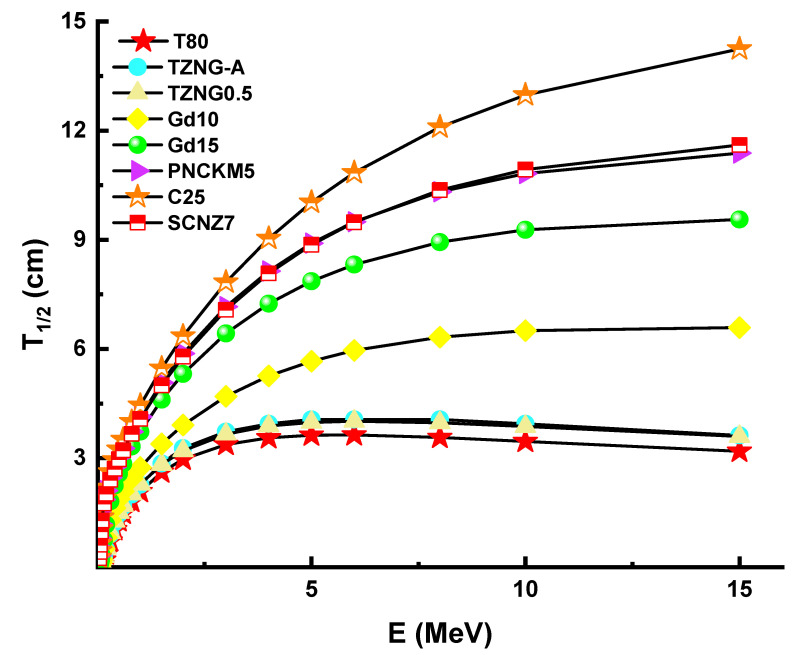
Half value layer comparison between some glasses and a T80 (10WO_3_ + 10MoO_3_ + 80TeO_2_) sample.

However, Compton scattering is the most observed type of photon–matter interaction at intermediate energies, although it is not the main interaction of total photon loss [26,27]. Consequently, the Compton area has some of the highest EBF values in the related energy region. Apart from regional variations in EBF levels, the T80 sample had the lowest EBF levels out of all T and S glass samples. In the context of the photon accumulation factor, it is referred to as the energy absorption build-up factor (EABF), with the primary parameter equal to the amount of energy gathered or deposited in the target molecule. EABF levels followed a similar path to EBF levels over the same period. Consequently, the T80 sample’s EABF minimum values were included. Finally, we have compared the gamma-ray shielding properties of the T80 sample with different types of shielding materials, such as TZNG-A [13], TZNG0.5 [14], Gd10 [15], Gd15 [16], PNCKM5 [17], C25 [18], SCNZ7 [19], Ordinary Concrete (OC), HSC, ILC, BMC, IC, and SSC [20]. This study sought to understand the overall performance of the T80 sample, in terms of the T_1/2_ values necessary to attenuate incoming gamma-ray photons from 0.015 to 15 MeV. As a result, the T_1/2_ values of the T80 samples were compared to those of many glass shields and standard/special kinds of concrete. The variation of T_1/2_ values for T80 and other glass shields as a function of incident photon energy is shown in Figure 10. 

**Figure 11:**
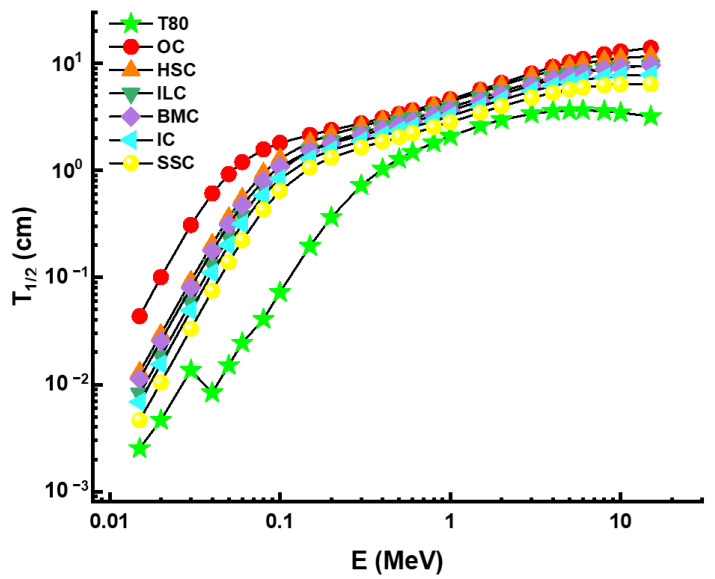
Half value layer comparison between some concretes and a T80 (10WO_3_ + 10MoO_3_ + 80TeO_2_) sample.

Our findings revealed that T80 has the lowest T_1/2_ values compared to TZNG-A, TZNG0.5, Gd10, Gd15, PNCKM5, C25, and SCNZ7 glasses, all of which have been examined before as possible glass shields. However, our data indicate that the T_1/2_ values of the TZNG0.5 sample are comparable to those of the T80 sample. This is explained by the similarities of the two glasses, with the T80 sample having a density of 5.61 g/cm^3^ and the TZNG0.5 sample having a density of 5.254 g/cm^3^. Finally, we will share the results of our comparison of the T_1/2_ values for T80 and other kinds of concrete. The findings are presented in Figure 11 as a function of incident photon energy. Overall, the T80 (10WO_3_ + 10MoO_3_ + 80TeO_2_) sample has been reported with the lowest T_1/2_ values. However, the difference is slightly more significant between 0.04 MeV and 0.3 MeV, indicating that the T80 sample may be the preferred candidate for some radiation facilities as an HMO glass shield, such as in diagnostic radiology and nuclear medicine [28], where the mentioned energy range is of interest for utilization.

## 4. Conclusions

One of the best examples that can be cited in the field of material science and its applications is the use of high quality and hardened glass materials as a shielding material in medical, industrial, and radiation research fields. According to the purpose and type of radiation field used, it is necessary to characterize each glass material in detail and determine its properties before use. Heavy metal oxide (HMO) glasses have always been an interesting glass group, in terms of its density and optical properties. This study aimed to provide important results on some novel HMO glasses containing heavy metal cations with a high ratio that are preferred in optoelectronics, due to their high transmittances in the visible and mid-IR region. These findings indicated that these new glasses had properties comparable to those of traditional materials. Additionally, it was found that the T80 (10WO_3_ + 10MoO_3_ + 80TeO_2_) sample’s gamma shielding capabilities, which were the greatest among the manufactured HMO glasses, were effective at greater levels than other materials, such as some glass shields and different types of concrete. However, it can be concluded that certain kinds of radiation, such as alpha, proton, and neutron, are worth further investigation. Furthermore, mechanical and thermal properties and elastic moduli are also worth further investigation, since durability and thermal conductivity are other important properties for any shielding material.

## Figures and Tables

**Table 1:** Chemical compositions (mol%) and density values of HMO glasses.

**Sample Code**	**WO_3_**	**MoO_3_**	**TeO_2_**	**Density (g/cm^3^)**
**T80**	10	10	80	5.61
**T70**	10	20	70	5.41
**T60**	10	30	60	5.29
**Sample Code**	**WO_3_**	**MoO_3_**	**Sb_2_O_3_**	**Density (g/cm^3^)**
**S80**	10	10	80	5.43
**S70**	10	20	70	5.29
**S60**	10	30	60	5.21

## Data Availability

The data presented in this study are available on request from the corresponding author.

## Supplementary Materials

The following are available online at https://www.mdpi.com/article/10.3390/ma14154330/s1, Table S1: (EBF and EABF) G-P fitting coefficients (b, c, a, Xk and d) of T80 glass sample., Table S2: (EBF and EABF) G-P fitting coefficients (b, c, a, Xk and d) of T70 glass sample., Table S3: (EBF and EABF) G-P fitting coefficients (b, c, a, Xk and d) of T60 glass sample., Table S4: (EBF and EABF) G-P fitting coefficients (b, c, a, Xk and d) of S80 glass sample., Table S5: (EBF and EABF) G-P fitting coefficients (b, c, a, Xk and d) of S70 glass sample., Table S6: (EBF and EABF) G-P fitting coefficients (b, c, a, Xk and d) of S60 glass sample.
