# Supplementary material for: Novel HMO-Glasses with Sb2O3 and TeO2 for Nuclear Radiation Shielding Purposes: A Comparative Analysis with Traditional and Novel Shields
Source: Materials (Basel). 2021 Aug 3;14(15):4330. doi: 10.3390/ma14154330 (PMC8348751; doi:10.3390/ma14154330)
Supplement: Supplementary file 1 [file materials-14-04330-s001.zip › materials-1314981-supplementary.pdf]

materials

MDPI

Supplementary Materials

Novel HMO-Glasses with Sb_2O_3 and TeO_2 for Nuclear Radiation Shielding Purposes: A Comparative Analysis with Traditional and Novel Shields

Ghada ALMisned [1], Huseyin Ozan Tekin [2,3], Shams A. M. Issa [4,5], Miray Çelikbilek Ersundu [6], Ali Erçin Ersundu [6], Gokhan Kilic [7], Hesham M. H. Zakaly [5,8,*] and Antoaneta Ene [9,*]

1 Department of Physics, College of Science, Princess Nourah Bint Abdulrahman University, Riyadh 11671, Saudi Arabia; gaalmisned@pnu.edu.sa
2 Department of Medical Diagnostic Imaging, College of Health Sciences, University of Sharjah, 27272 Sharjah, United Arab Emirates; tekin765@gmail.com
3 Medical Radiation Research Center (USMERA), Uskudar University, 34672 Istanbul, Turkey
4 Physics Department, Faculty of Science, University of Tabuk, Tabuk 71451, Saudi Arabia; shams_issa@yahoo.com
5 Physics Department, Faculty of Science, Al-Azhar University, Assiut 71524, Egypt
6 Glass Research and Development Laboratory, Department of Metallurgical and Materials Engineering, Faculty of Chemical and Metallurgical Engineering, Yildiz Technical University, 34220 Istanbul, Turkey; miraycelikbilek@gmail.com (M.Ç.E.); ersundu@gmail.com (A.E.E.)
7 Department of Physics, Faculty of Science and Letters, Eskisehir Osmangazi University, 26040 Eskisehir, Turkey; gkilic@ogu.edu.tr
8 Institute of Physics and Technology, Ural Federal University, 620000 Ekaterinburg, Russia
9 INPOLDE Research Center, Department of Chemistry, Faculty of Sciences and Environment, Physics and Environment, Dunarea de Jos University of Galati, 47 Domneasca Street, 800008 Galati, Romania
* Correspondence: h.m.zakaly@gmail.com or h.m.zakaly@azhar.edu.eg (H.M.H.Z.); Antoaneta.Ene@ugal.ro (A.E.)

Citation: ALMisned, G.; Tekin, H.O.; Issa, S.A.M.; Ersundu, M.Ç.; Ersundu, A.E.; Kilic, G.; Zakaly, H.M.H. Novel HMO-Glasses with Sb_2O_3 and TeO_2 for Nuclear Radiation Shielding Purposes: A Comparative Analysis with Traditional and Novel Shields. *Materials* **2021**, *14*, 4330. https://doi.org/10.3390/ma14154330

Academic Editor: Gerhard Wilde

Received: 10 July 2021
Accepted: 31 July 2021
Published: 3 August 2021

Publisher's Note: MDPI stays neutral with regard to jurisdictional claims in published maps and institutional affiliations.

Copyright: © 2021 by the author. Licensee MDPI, Basel, Switzerland. This article is an open access article distributed under the terms and conditions of the Creative Commons Attribution (CC BY) license (http://creativecommons.org/licenses/by/4.0/).

Table S1. (EBF and EABF) G–P fitting coefficients (b, c, a, Xk and d) of T80 glass sample.

Energy (MeV)	Z_{eq}	G–P Fitting Parameters for EBF					G–P Fitting Parameters for EABF				
		a	b	c	d	X_k	a	b	c	d	X_k
0.015	25.94	−0.549	1.004	1.553	0.350	5.612	−0.549	1.004	1.553	0.350	5.635
0.020	27.56	0.697	1.010	0.116	−0.854	11.064	0.385	1.008	0.243	−0.410	15.224
0.030	28.00	0.200	1.023	0.364	−0.137	17.583	0.249	1.020	0.353	−0.182	13.713
0.040	44.00	0.090	3.843	0.562	−0.057	24.158	0.102	1.535	0.571	−0.040	20.576
0.050	44.41	−0.126	3.230	0.183	−0.038	13.507	−0.015	1.465	0.193	0.007	10.978
0.060	44.69	0.701	2.599	0.086	−0.115	13.070	0.487	1.413	0.106	−0.104	17.135
0.080	49.35	0.808	1.797	0.018	−0.160	15.371	0.721	1.386	0.038	−0.227	14.225
0.100	49.75	0.676	1.389	0.063	−0.317	13.789	0.649	1.333	0.074	−0.353	13.421
0.150	50.31	0.283	1.218	0.322	−0.158	13.940	0.434	1.438	0.178	−0.238	13.855
0.200	50.64	0.173	1.255	0.495	−0.093	14.579	0.333	1.614	0.276	−0.194	13.912
0.300	51.03	0.110	1.390	0.637	−0.052	14.169	0.215	1.900	0.439	−0.123	13.776
0.400	51.25	0.069	1.509	0.774	−0.044	14.136	0.161	2.226	0.571	−0.113	13.868
0.500	51.40	0.047	1.584	0.857	−0.036	14.068	0.114	2.309	0.687	−0.088	13.872
0.600	51.50	0.028	1.620	0.919	−0.026	13.973	0.089	2.349	0.755	−0.074	13.726
0.800	51.60	0.012	1.660	0.983	−0.020	13.987	0.059	2.315	0.846	−0.057	13.625
1.000	51.64	0.004	1.665	1.015	−0.018	13.383	0.043	2.238	0.896	−0.049	13.507
1.500	50.83	−0.020	1.566	1.116	−0.005	14.218	0.008	1.928	1.021	−0.027	13.645
2.000	48.59	−0.017	1.563	1.111	−0.008	13.021	0.016	1.849	1.002	−0.036	13.112
3.000	45.37	0.002	1.542	1.062	−0.030	12.906	0.040	1.717	0.942	−0.063	13.237
4.000	43.98	0.016	1.496	1.026	−0.043	13.379	0.056	1.596	0.902	−0.078	13.552
5.000	43.25	0.046	1.514	0.945	−0.071	13.607	0.085	1.568	0.832	−0.104	13.798
6.000	42.81	0.057	1.495	0.922	−0.080	13.831	0.097	1.514	0.809	−0.115	14.011
8.000	42.23	0.080	1.531	0.879	−0.098	14.150	0.111	1.480	0.793	−0.125	14.292
10.000	41.93	0.059	1.501	0.965	−0.078	14.209	0.087	1.412	0.878	−0.101	14.350
15.000	41.75	0.035	1.567	1.116	−0.060	14.165	0.063	1.402	1.017	−0.084	14.289

Table S2. (EBF and EABF) G–P fitting coefficients (b, c, a, Xk and d) of T70 glass sample.

Energy (MeV)	Z_{eq}	G–P Fitting Parameters for EBF					G–P Fitting Parameters for EABF				
		a	b	c	d	X_k	a	b	c	d	X_k
0.015	25.49	−0.510	1.004	1.488	0.335	5.709	−0.509	1.004	1.487	0.334	5.913
0.020	28.49	0.741	1.009	0.107	−0.989	10.879	0.422	1.007	0.235	−0.475	13.525
0.030	29.04	0.205	1.030	0.362	−0.052	12.076	0.246	1.019	0.373	−0.177	11.323
0.040	43.03	0.089	3.890	0.447	−0.045	23.781	0.104	1.513	0.451	−0.042	21.841
0.050	43.45	−0.201	3.182	0.126	−0.001	12.899	−0.070	1.433	0.137	0.052	9.926
0.060	43.74	0.840	2.533	0.057	−0.126	14.608	0.595	1.383	0.077	−0.134	16.366
0.080	48.56	0.804	1.783	0.020	−0.170	15.274	0.706	1.377	0.042	−0.227	14.206
0.100	48.98	0.641	1.368	0.085	−0.302	13.785	0.620	1.320	0.091	−0.336	13.455
0.150	49.58	0.274	1.220	0.334	−0.152	13.973	0.421	1.438	0.188	−0.232	13.872
0.200	49.92	0.171	1.263	0.500	−0.092	14.617	0.326	1.630	0.283	−0.191	13.921
0.300	50.34	0.107	1.400	0.644	−0.051	14.210	0.208	1.911	0.451	−0.118	13.806
0.400	50.57	0.067	1.521	0.782	−0.043	14.138	0.156	2.244	0.581	−0.110	13.869
0.500	50.73	0.045	1.596	0.863	−0.036	14.064	0.111	2.335	0.696	−0.086	13.871
0.600	50.83	0.026	1.631	0.926	−0.026	13.994	0.086	2.369	0.764	−0.073	13.727
0.800	50.94	0.010	1.670	0.990	−0.019	14.029	0.056	2.329	0.854	−0.056	13.627
1.000	50.99	0.003	1.673	1.021	−0.017	13.402	0.041	2.246	0.903	−0.048	13.504
1.500	50.11	−0.021	1.569	1.121	−0.005	14.289	0.005	1.918	1.033	−0.024	13.658
2.000	47.70	−0.017	1.568	1.113	−0.008	12.970	0.014	1.849	1.008	−0.035	13.106
3.000	44.31	0.001	1.547	1.063	−0.030	12.886	0.038	1.718	0.946	−0.061	13.228
4.000	42.90	0.016	1.501	1.024	−0.043	13.369	0.055	1.598	0.904	−0.076	13.535
5.000	42.14	0.046	1.518	0.942	−0.070	13.592	0.084	1.570	0.833	−0.103	13.782
6.000	41.68	0.056	1.496	0.923	−0.079	13.808	0.094	1.511	0.815	−0.111	14.006
8.000	41.10	0.078	1.521	0.883	−0.096	14.112	0.107	1.469	0.802	−0.120	14.276
10.000	40.81	0.058	1.485	0.965	−0.077	14.187	0.083	1.398	0.884	−0.098	14.348
15.000	40.61	0.037	1.538	1.104	−0.060	14.189	0.062	1.382	1.011	−0.083	14.328

Table S3. (EBF and EABF) G–P fitting coefficients (b, c, a, Xk and d) of T60 glass sample.

Energy (MeV)	Z_{eq}	G–P Fitting Parameters for EBF					G–P Fitting Parameters for EABF				
		a	b	c	d	X_k	a	b	c	d	X_k
0.015	25.01	−0.469	1.004	1.418	0.319	5.811	−0.467	1.004	1.417	0.317	6.208
0.020	29.35	0.740	1.059	0.166	−1.027	10.862	0.428	1.013	0.289	−0.494	12.790
0.030	30.01	0.196	1.261	0.426	−0.066	13.853	0.234	1.063	0.437	−0.172	12.801
0.040	42.04	0.088	3.939	0.327	−0.033	23.388	0.107	1.490	0.326	−0.045	23.161
0.050	42.46	−0.280	3.132	0.066	0.038	12.260	−0.127	1.399	0.079	0.100	8.820
0.060	42.76	0.987	2.463	0.028	−0.138	16.240	0.709	1.352	0.047	−0.166	15.550
0.080	47.75	0.799	1.769	0.021	−0.180	15.172	0.691	1.368	0.046	−0.227	14.187
0.100	48.18	0.604	1.345	0.108	−0.287	13.782	0.589	1.307	0.108	−0.319	13.491
0.150	48.81	0.265	1.222	0.349	−0.146	14.015	0.409	1.439	0.200	−0.225	13.893
0.200	49.17	0.171	1.281	0.501	−0.093	14.588	0.325	1.687	0.285	−0.192	13.931
0.300	49.61	0.105	1.413	0.651	−0.050	14.242	0.203	1.938	0.461	−0.116	13.831
0.400	49.87	0.065	1.534	0.790	−0.042	14.141	0.151	2.266	0.592	−0.108	13.871
0.500	50.04	0.043	1.609	0.871	−0.036	14.060	0.107	2.362	0.705	−0.085	13.870
0.600	50.14	0.024	1.643	0.934	−0.025	14.016	0.083	2.392	0.773	−0.071	13.730
0.800	50.25	0.009	1.679	0.996	−0.018	14.073	0.054	2.343	0.862	−0.055	13.629
1.000	50.30	0.002	1.681	1.026	−0.016	13.421	0.039	2.255	0.911	−0.047	13.501
1.500	49.35	−0.022	1.574	1.125	−0.004	13.763	0.003	1.919	1.039	−0.023	13.646
2.000	46.77	−0.018	1.573	1.115	−0.007	12.916	0.012	1.849	1.014	−0.033	13.101
3.000	43.23	0.001	1.551	1.064	−0.029	12.865	0.036	1.719	0.951	−0.059	13.220
4.000	41.79	0.016	1.506	1.023	−0.043	13.354	0.053	1.599	0.908	−0.074	13.528
5.000	41.02	0.044	1.516	0.946	−0.068	13.569	0.080	1.562	0.843	−0.098	13.804
6.000	40.55	0.054	1.490	0.928	−0.076	13.764	0.089	1.501	0.827	−0.106	14.028
8.000	39.98	0.074	1.507	0.889	−0.093	14.063	0.101	1.455	0.814	−0.115	14.258
10.000	39.69	0.056	1.469	0.965	−0.075	14.164	0.080	1.384	0.890	−0.094	14.347
15.000	39.50	0.038	1.510	1.093	−0.061	14.213	0.062	1.362	1.006	−0.081	14.368

Table S4. EBF and EABF) G–P fitting coefficients (b, c, a, Xk and d) of S80 glass sample.

Energy (MeV)	Z_{eq}	G–P Fitting Parameters for EBF					G–P Fitting Parameters for EABF				
		a	b	c	d	X_k	a	b	c	d	X_k
0.015	25.49	−0.511	1.004	1.489	0.336	5.707	−0.510	1.004	1.488	0.334	5.909
0.020	26.54	0.647	1.011	0.125	−0.700	11.275	0.342	1.009	0.253	−0.336	17.162
0.030	26.86	0.194	1.026	0.370	−0.238	24.197	0.251	1.024	0.333	−0.187	16.620
0.040	45.10	0.092	3.791	0.689	−0.070	24.575	0.099	1.559	0.704	−0.037	19.178
0.050	45.41	−0.050	3.278	0.241	−0.075	14.125	0.040	1.498	0.249	−0.039	12.048
0.060	45.63	0.566	2.663	0.113	−0.104	11.573	0.382	1.441	0.134	−0.075	17.883
0.080	48.53	0.803	1.783	0.020	−0.170	15.270	0.706	1.377	0.042	−0.227	14.206
0.100	48.83	0.634	1.364	0.089	−0.299	13.785	0.614	1.318	0.094	−0.333	13.462
0.150	49.25	0.270	1.221	0.340	−0.150	13.991	0.416	1.439	0.193	−0.229	13.881
0.200	49.49	0.171	1.274	0.501	−0.092	14.601	0.326	1.663	0.284	−0.191	13.926
0.300	49.78	0.105	1.410	0.649	−0.050	14.237	0.204	1.929	0.459	−0.116	13.826
0.400	49.95	0.065	1.532	0.789	−0.042	14.140	0.152	2.262	0.591	−0.108	13.870
0.500	50.06	0.043	1.609	0.870	−0.036	14.060	0.107	2.362	0.705	−0.085	13.870
0.600	50.12	0.024	1.643	0.934	−0.025	14.016	0.083	2.392	0.773	−0.071	13.730
0.800	50.20	0.008	1.680	0.997	−0.018	14.077	0.054	2.344	0.863	−0.055	13.629
1.000	50.22	0.001	1.682	1.027	−0.016	13.424	0.039	2.256	0.912	−0.047	13.501
1.500	49.56	−0.021	1.573	1.124	−0.004	13.937	0.003	1.918	1.038	−0.024	13.650
2.000	47.78	−0.017	1.568	1.113	−0.008	12.975	0.015	1.849	1.007	−0.035	13.107
3.000	45.32	0.002	1.542	1.062	−0.030	12.905	0.040	1.717	0.942	−0.063	13.236
4.000	44.26	0.016	1.494	1.027	−0.043	13.381	0.057	1.595	0.901	−0.078	13.556
5.000	43.69	0.046	1.512	0.945	−0.071	13.613	0.086	1.567	0.831	−0.105	13.805
6.000	43.35	0.057	1.494	0.923	−0.080	13.838	0.098	1.514	0.808	−0.116	14.018
8.000	42.91	0.080	1.530	0.882	−0.098	14.151	0.111	1.480	0.794	−0.125	14.296
10.000	42.66	0.058	1.502	0.971	−0.078	14.207	0.087	1.413	0.881	−0.102	14.345
15.000	42.54	0.034	1.574	1.125	−0.060	14.142	0.063	1.407	1.023	−0.085	14.266

Table S5. (EBF and EABF) G–P fitting coefficients (b, c, a, Xk and d) of S70 glass sample.

Energy (MeV)	Z_{eq}	G–P Fitting Parameters for EBF					G–P Fitting Parameters for EABF				
		a	b	c	d	X_k	a	b	c	d	X_k
0.015	25.23	−0.488	1.004	1.450	0.327	5.765	−0.486	1.004	1.449	0.325	6.075
0.020	27.28	0.683	1.011	0.118	−0.812	11.122	0.373	1.008	0.246	−0.390	15.753
0.030	27.69	0.199	1.023	0.365	−0.164	19.341	0.249	1.021	0.348	−0.183	14.486
0.040	44.42	0.091	3.823	0.611	−0.062	24.318	0.101	1.544	0.622	−0.039	20.039
0.050	44.74	−0.100	3.246	0.202	−0.051	13.717	0.004	1.476	0.212	−0.009	11.343
0.060	44.97	0.659	2.619	0.094	−0.112	12.610	0.455	1.422	0.115	−0.095	17.365
0.080	48.08	0.801	1.775	0.021	−0.176	15.214	0.697	1.372	0.045	−0.227	14.195
0.100	48.40	0.614	1.352	0.101	−0.291	13.783	0.597	1.310	0.103	−0.323	13.481
0.150	48.85	0.265	1.222	0.348	−0.147	14.013	0.409	1.439	0.200	−0.225	13.892
0.200	49.11	0.171	1.283	0.501	−0.093	14.586	0.325	1.692	0.285	−0.192	13.931
0.300	49.41	0.104	1.418	0.653	−0.050	14.248	0.202	1.949	0.463	−0.115	13.836
0.400	49.59	0.064	1.539	0.793	−0.042	14.142	0.150	2.280	0.596	−0.107	13.872
0.500	49.71	0.042	1.615	0.875	−0.035	14.069	0.106	2.375	0.710	−0.084	13.871
0.600	49.78	0.023	1.649	0.937	−0.025	14.018	0.081	2.402	0.778	−0.071	13.731
0.800	49.87	0.008	1.685	1.000	−0.018	14.089	0.053	2.350	0.867	−0.054	13.630
1.000	49.90	0.001	1.686	1.030	−0.016	13.430	0.038	2.260	0.915	−0.046	13.501
1.500	49.18	−0.022	1.575	1.126	−0.004	13.622	0.003	1.919	1.040	−0.023	13.642
2.000	47.26	−0.018	1.571	1.114	−0.008	12.945	0.014	1.849	1.011	−0.034	13.104
3.000	44.65	0.001	1.545	1.063	−0.030	12.893	0.039	1.718	0.945	−0.062	13.231
4.000	43.55	0.016	1.498	1.025	−0.043	13.375	0.056	1.597	0.903	−0.077	13.545
5.000	42.95	0.046	1.515	0.944	−0.071	13.603	0.085	1.568	0.832	−0.104	13.794
6.000	42.60	0.057	1.496	0.922	−0.080	13.828	0.097	1.514	0.810	−0.114	14.008
8.000	42.14	0.080	1.531	0.879	−0.098	14.150	0.111	1.480	0.792	−0.125	14.291
10.000	41.88	0.059	1.500	0.965	−0.078	14.208	0.087	1.412	0.879	−0.101	14.350
15.000	41.76	0.035	1.567	1.116	−0.060	14.165	0.063	1.402	1.017	−0.084	14.288

Table S6. (EBF and EABF) G–P fitting coefficients (b, c, a, Xk and d) of S60 glass sample.

Energy (MeV)	Z_{eq}	G–P Fitting Parameters for EBF					G–P Fitting Parameters for EABF				
		a	b	c	d	X_k	a	b	c	d	X_k
0.015	24.92	−0.461	1.004	1.406	0.316	5.830	−0.459	1.004	1.404	0.314	6.263
0.020	28.04	0.720	1.010	0.111	−0.924	10.968	0.404	1.007	0.239	−0.444	14.339
0.030	28.55	0.203	1.021	0.361	−0.089	14.484	0.248	1.018	0.363	−0.179	12.352
0.040	43.65	0.090	3.860	0.520	−0.053	24.021	0.103	1.527	0.528	−0.041	21.037
0.050	43.99	−0.159	3.209	0.158	−0.022	13.243	−0.039	1.451	0.169	0.027	10.522
0.060	44.23	0.767	2.567	0.072	−0.121	13.805	0.539	1.399	0.092	−0.118	16.767
0.080	47.58	0.798	1.766	0.022	−0.182	15.151	0.687	1.367	0.047	−0.227	14.183
0.100	47.91	0.591	1.338	0.116	−0.281	13.780	0.578	1.302	0.114	−0.312	13.503
0.150	48.40	0.259	1.223	0.357	−0.143	14.038	0.402	1.440	0.207	−0.221	13.905
0.200	48.67	0.171	1.294	0.502	−0.093	14.569	0.325	1.725	0.287	−0.192	13.937
0.300	49.00	0.103	1.426	0.657	−0.049	14.260	0.200	1.971	0.468	−0.114	13.848
0.400	49.20	0.062	1.548	0.798	−0.042	14.144	0.148	2.299	0.602	−0.106	13.874
0.500	49.33	0.041	1.623	0.879	−0.035	14.081	0.104	2.390	0.716	−0.083	13.872
0.600	49.40	0.023	1.655	0.941	−0.024	14.014	0.080	2.414	0.783	−0.070	13.732
0.800	49.50	0.007	1.690	1.003	−0.018	14.086	0.051	2.357	0.871	−0.054	13.632
1.000	49.52	0.000	1.690	1.032	−0.016	13.430	0.037	2.265	0.919	−0.045	13.503
1.500	48.75	−0.022	1.578	1.128	−0.004	13.260	0.002	1.920	1.043	−0.023	13.632
2.000	46.68	−0.018	1.574	1.115	−0.007	12.911	0.012	1.849	1.015	−0.033	13.100
3.000	43.90	0.001	1.548	1.063	−0.030	12.878	0.037	1.718	0.948	−0.061	13.225
4.000	42.76	0.016	1.501	1.024	−0.043	13.367	0.055	1.598	0.904	−0.076	13.532
5.000	42.14	0.046	1.518	0.942	−0.070	13.592	0.084	1.570	0.833	−0.103	13.782
6.000	41.75	0.056	1.496	0.922	−0.079	13.811	0.095	1.512	0.814	−0.112	14.005
8.000	41.28	0.078	1.523	0.882	−0.096	14.120	0.107	1.471	0.800	−0.121	14.279
10.000	41.03	0.058	1.488	0.965	−0.077	14.191	0.084	1.401	0.883	−0.098	14.349
15.000	40.89	0.036	1.545	1.107	−0.060	14.183	0.063	1.387	1.013	−0.083	14.318
